# Genome-scale metabolic model analysis of Korean *Campylobacter jejuni* isolates reveals lineage-level metabolic structure

**DOI:** 10.1186/s44342-026-00086-3

**Published:** 2026-11-02

**Authors:** Myeong-Won Jo, Seung-Hyun Jung, Jeong-Ih Shin, Yeun-Jun Chung

**Affiliations:** 1 Department of Medical Sciences, Graduate School of the Catholic University of Korea, Seoul, 06591 Republic of Korea; 2 Department of Microbiology, College of Medicine, The Catholic University of Korea, Seoul, 06591 Republic of Korea; 3 Catholic Research Institute for Human Genome Polymorphism, Precision Medicine Research Center, College of Medicine, The Catholic University of Korea, Seoul, 06591 Republic of Korea; 4 Department of Biochemistry, College of Medicine, The Catholic University of Korea, Seoul, 06591 Republic of Korea

**Keywords:** *Campylobacter jejuni*, Genome-scale metabolic model, Flux balance analysis, Clonal complex, Substrate utilization

## Abstract

Genome-scale metabolic models (GSMMs) of *Campylobacter jejuni* (*C. jejuni*) have been studied mainly for reference strains, and their characteristics at the lineage level remain poorly understood. We reconstructed GSMMs for 86 *C. jejuni* isolates collected in Korea using gapseq and analyzed them together with in vitro substrate utilization data for seven substrates measured on the same isolates. Strain-specific GSMMs clustered by clonal complex (CC): in all nine CCs with ≥ 2 isolates the mean within-CC Jaccard distance was smaller than the mean between-CC distance; across all strain pairs the median distance was 0.082 within CCs and 0.130 between CCs (*P* < 0.001). In the in vitro phenotypes, only five of the nine CCs showed smaller within- than between-CC distances, and the GSMM and in vitro distances were only weakly correlated (Mantel *r* = 0.13, *P* = 0.03). In an exploratory comparison of two lineages with contrasting profiles, CC-443 (*n* = 7) showed negative mean *z*-scores for all seven substrates, whereas CC-658 (*n* = 4) showed positive mean *z*-scores for all seven, the highest being sulfite (z = + 0.95). In GSMMs, CC-443 lacked reactions for the monoamine/polyamine oxidase family and pantothenate/CoA biosynthesis, whereas CC-658 was enriched in fatty acid/phospholipid biosynthesis, sialic acid metabolism, and anaerobic sulfur respiration. Anaerobic sulfur respiration-related reactions were more prevalent in CC-658 (75%) than in the other CCs (11%), consistent with its high in vitro sulfite utilization; this difference was retained when the comparison was restricted to chicken isolates (0% in CC-443 vs. 100% in CC-658). In silico growth simulations under two gut-mimicking diets (Western and high-fiber) showed consistently lower predicted growth for CC-443 than for CC-658 under both conditions (exact permutation test, *P* = 0.048), although the eleven models yielded only four distinct predicted values under each diet, which limits the strength of this comparison. These results indicate that metabolic modeling of *C. jejuni* benefits from considering lineage-level diversity rather than relying on a single reference-strain GSMM.

**Supplementary Information:**

The online version contains supplementary material available at https://doi.org/10.1186/s44342-026-00086-3.

## Background

*Campylobacter jejuni* (*C. jejuni*) is one of the leading bacterial causes of foodborne gastroenteritis worldwide [1], and numerous studies have investigated the prevalence of *Campylobacter* contamination in poultry and meat products in Korea [2]. Unlike many enteric bacteria, *C. jejuni* has a limited capacity for carbohydrate catabolism and primarily relies on amino acids and peptides as carbon and energy sources. In particular, serine, aspartate, asparagine, glutamine, glutamate, and proline are preferentially catabolized to support energy production and biosynthesis [3]. In addition, *C. jejuni* exploits metabolites supplied by the host microbiota for respiration, using compounds such as formate, hydrogen, sulfite, and gluconate as electron donors [3]. The ability to utilize these substrates varies among strains [3], which in turn influences their capacity to colonize different host environments [4].

Genome-scale metabolic models (GSMMs) provide a systems-level framework for characterizing the metabolic capabilities of microorganisms [5]. Automated reconstruction pipelines such as gapseq enable strain-specific GSMMs to be generated directly from whole-genome sequences [6], facilitating flux balance analysis (FBA) [7] as well as comparative analyses of metabolic reaction repertoires across multiple strains [8, 9]. Previous GSMM-based studies of *C. jejuni* have focused primarily on reference strains [10–12]. In contrast, relatively few studies have investigated lineage-specific metabolic diversity among clinical and food isolates or directly compared GSMM predictions with experimentally measured in vitro phenotypes.

In this study, we reconstructed GSMMs for 86 *C. jejuni* isolates from Korea and integrated these models with in vitro substrate utilization data measured for the same strains using seven substrates (lactate, formate, sulfite, gluconate, serine, proline, and asparagine). We aimed to address two primary questions: (i) whether automatically reconstructed GSMMs capture lineage-specific metabolic characteristics defined by clonal complexes (CCs), and (ii) to what extent in vitro phenotypic variation is reflected in GSMM reaction content. Building on these, we examined the GSMM-level features associated with the contrasting phenotypes of two representative lineages, CC-443 and CC-658, and evaluated whether their phenotypic differences could be reproduced by GSMM-based in silico growth simulations under gut-mimicking dietary conditions.

## Materials and methods

### Bacterial isolates and clinical metadata

A total of 86 Korean *C. jejuni* isolates analyzed in this study were collected between 2015 and 2023 from raw chicken meat (*n* = 70, 81%) and human fecal samples (*n* = 16, 19%) across seven regions of Korea (Chungcheongbuk-do, Chungcheongnam-do, Gyeonggi-do, Gangwon-do, Gyeongsangnam-do, Seoul, and Incheon). All isolates were stored at − 80 °C in 20% glycerol stocks and were cultured on Preston agar at 42 °C for 24 h under microaerobic conditions (5% O_2_ and 10% CO_2_) before phenotypic experiments. The isolation source and year of collection for each isolate were recorded and are provided (Supplemental Table S1). This study was exempt from institutional review board approval by the Institutional Review Board of the Catholic University of Korea (No. MC24SASI0019). All human-derived isolates and associated metadata were analyzed in a fully de-identified manner.

### Whole-genome sequencing, assembly, and annotation

Genomic DNA was extracted from pure bacterial cultures using the iDetect gDNA Prep Kit for Microbes (ConnectaGen, Hanam, South Korea). Whole-genome sequencing was performed on the Illumina platform using the TruSeq Nano DNA Sample Preparation Kit (Illumina, San Diego, CA, USA). Raw sequencing reads were quality-filtered using Trimmomatic and de novo assembled with SPAdes (version 3.9.0) [13]. Assembly quality was assessed using QUAST (version 5.3.0) [14]. Genome annotation was performed using Prokka (version 1.14.5) [15].

The species identity of each isolate was confirmed using Kraken2 (version 2.1.4) [16]. Multilocus sequence typing (MLST) was performed using the mlst software and CC was assigned based on the *Campylobacter jejuni/coli* scheme available from PubMLST [17].

### In vitro substrate utilization assay

Substrate utilization was evaluated using seven compounds. Sodium L-lactate, L-serine, L-proline, and L-asparagine were used as carbon sources [4, 18–20], whereas sodium formate, sodium sulfite, and sodium D-gluconate were used as alternative electron donors [21, 22].

For each strain-substrate combination, bacterial cultures grown in Mueller–Hinton broth were adjusted to an optical density at 600 nm (OD_600_) of 0.1, dispensed into a 96-well plate at 190 μL per well, and supplemented with 10 μL of a 400 mM (20 ×) substrate stock solution (final substrate concentration, 20 mM; final volume, 200 μL). Each strain-substrate combination was tested in four technical replicates. For each strain, three conditions were assayed in parallel: (i) Mueller–Hinton (MH) broth supplemented with one of the seven test substrates (lactate, formate, sulfite, gluconate, serine, proline, or asparagine), (ii) the same MH broth without substrate supplementation (no-substrate control), and (iii) uninoculated MH broth (blank control). Plates were incubated statically at 42 °C under microaerobic conditions (5% O_2_ and 10% CO_2_), and OD_600_ was measured after 24 h using a BioTek Synergy HTX multimode microplate reader.

Substrate-dependent growth was expressed as the background-corrected fold change (fc) of the substrate-supplemented condition relative to the no-substrate control and was calculated as follows: fc = (OD substrate − OD background)/(OD no-substrate − OD background) (Supplemental Table S2). If the coefficient of variation (CV) among the four technical replicates exceeded 15%, the replicate with the largest deviation from the mean was excluded, and the mean fc was calculated using the remaining replicates. The mean fc values were then normalized to *z*-scores across 86 isolates for each substrate according to the following equation: *z* = (fc − mean of all isolates)/standard deviation (SD) of all isolates, where the SD was calculated over the 86 isolates without a degrees-of-freedom correction. The *z*-score represents the deviation of each value from the overall mean of all isolates in units of standard deviation, allowing direct comparison among substrates despite differences in their scales.

### Genome-scale metabolic model reconstruction

GSMMs were reconstructed for all 86 isolates using gapseq v1.2 (commit 6c2ff0e9; doall, default parameters) with the ModelSEED reaction database. Each model was saved in Systems Biology Markup Language (SBML) format and imported into Python using the COBRA library. Reaction content was extracted as a binary presence/absence matrix consisting of 2313 unique reactions across 86 isolates, with an average of 1505 reactions per model (range, 1391–1569) (Supplemental Table S3).

### Lineage analysis and statistical tests

The distance between GSMM reaction compositions was calculated as the Jaccard distance based on binary presence/absence vectors. For each pair of strains, reaction compositions were represented as binary vectors over the pan-reactome consisting of 2313 unique reactions. Each reaction was classified as present in both strains (*a*), present in only one strain (*b* or *c*), or absent in both strains (*d*). The Jaccard distance was defined as (*d*_*J*_ = (*b* + *c*)/(*a* + *b* + *c*)). Pairwise Jaccard distances were computed for all 3655 strain pairs (731 within-CC and 2924 between-CC). Because pairwise distances are not independent, CC-level structure was assessed using permutation-based methods (PERMANOVA and the Mantel test, 9999 permutations). For descriptive comparison, the median within-CC and between-CC distances were also reported. Because each isolate contributes to multiple pairs, 95% confidence intervals for the mean within- and between-CC distances were obtained by isolate-level bootstrapping (2000 resamples, with isolates rather than pairs resampled with replacement; Supplemental Tables S10 and S11). We also assessed whether assembly or model quality contributed to the observed distances. For each isolate we compiled genome completeness and contamination (CheckM2, version 1.1.0), contig count, largest contig, genome size, GC content, N50, L50, the percentage of called cgMLST alleles, gene count from the pan-genome analysis, and the number of gap-filled reactions, defined as the difference in reaction number between the gapseq draft and final models (Supplemental Table S5). Each metric was related to reaction count by Spearman correlation. The metrics were then standardized to zero mean and unit variance to build a Euclidean quality-distance matrix, and the association between reaction-content distance and CC membership was re-evaluated by partial Mantel test controlling for this matrix (9999 permutations).

The in vitro phenotypic distance was calculated as the Euclidean distance between the *z-*score-normalized fold-change vectors for the seven substrates. CC-level structure in the in vitro phenotypes was assessed using the same permutation-based approach (PERMANOVA and Mantel test). Enrichment of lineage-specific reactions for each CC was evaluated using Fisher’s exact test based on reaction presence/absence and CC membership. For each reaction, the difference in the proportion of strains carrying the reaction between isolates within and outside the corresponding CC, together with the Fisher’s exact test *p*-value, was calculated. Reactions with an absolute difference in reaction frequency of at least 50 percentage points and a nominal *P* < 0.01 were considered lineage-associated; *p*-values were additionally adjusted using the Benjamini–Hochberg false discovery rate (FDR), and FDR-adjusted results are reported alongside. The relationship between the two distance matrices was evaluated using the Mantel test (Spearman correlation, 9999 permutations). In addition to comparing each CC with all other isolates, reaction content was compared directly between CC-443 and CC-658 by Fisher’s exact test, both for all isolates and for chicken isolates only (Supplemental Table S7).

Differentially distributed reactions were grouped into functional pathway categories by keyword matching of the reaction names in the gapseq models. Major functional categories, including pantothenate/CoA biosynthesis, Guanosine diphosphate (GDP)-mannose/fucose biosynthesis, anaerobic sulfur respiration, sialic acid biosynthesis, and fatty acid biosynthesis, were independently validated against Kyoto Encyclopedia of Genes and Genomes (KEGG) pathway and module annotations using KEGG Mapper [23].

### In silico growth simulation under gut-mimicking dietary conditions

To evaluate the metabolic capabilities of the GSMMs reconstructed for all 86 isolates under more complex nutritional environments, FBA simulations were performed under two representative nutritional conditions adapted from the Western and high-fiber diet concepts described by Magnusdottir et al. [24]; the corresponding metabolites were mapped to ModelSEED exchange reactions (Supplemental Table S4). The Western diet was designed to represent an environment rich in simple sugars, lipids, and amino acids but low in dietary fiber, and consisted of major monosaccharides and disaccharides (glucose, fructose, lactose, maltose, sucrose, galactose, and mannose), short-chain fatty acids (acetate, propionate, and butyrate), all 20 amino acids, B-complex vitamins including pantothenate, host-derived mucin glycans (N-acetylglucosamine, sialic acid, and fucose), multiple sulfur sources (sulfate, sulfite, and thiosulfate), osmoprotectants and lipid precursors (glycerol, choline, and glycine betaine), and unlimited inorganic ions. Predicted growth rates were compared between lineages using a two-sided exact permutation test. Because the predictions differed only at solver precision, values were rounded to ten decimal places before ranking, tied values received the average rank, and all possible group assignments were enumerated. The two-sided *P* value was defined as the proportion of assignments whose rank sum deviated from the null expectation by at least as much as the observed value in absolute terms. Cohen’s *d* was computed as a descriptive measure of effect size with a bootstrap interval (10,000 resamples) computed from the unrounded predictions; resamples in which the pooled standard deviation fell below 1e−12 were treated as undefined and excluded, and their proportion is reported. Per-strain predictions are provided in Supplemental Table S8.

The high-fiber diet was designed to represent a dietary fiber fermentation environment. Compared with the Western diet, the uptake rates of simple sugars (e.g., glucose) and amino acids were reduced, whereas the availability of the fiber degradation product cellobiose and the fermentation products short-chain fatty acids (acetate, propionate, and butyrate), lactate, and formate was increased. The exact exchange-reaction bounds for both diets are provided in Supplemental Table S4. Microaerobic conditions were simulated by setting the upper bound of oxygen uptake to 5 mmol/gDW/h. FBA was performed using the cobra library (version 0.31.1) in Python 3.11.5, with the biomass production reaction as the objective function. The theoretical maximum growth rate (*μ*, 1/h) attainable by each strain under the two dietary conditions was then calculated.

### Reproducibility

The analysis pipeline and figure-generation scripts have been uploaded to the GitHub repository (https://github.com/Jeong-Ih-Shin/cjejuni-gem-lineage-). The 86 strain-specific genome-scale metabolic models (SBML format) are available from Zenodo (https://doi.org/10.5281/zenodo.21886583). The whole-genome sequencing raw reads of the 86 isolates used in this study have been deposited in the National Center for Biotechnology Information (NCBI) Sequence Read Archive (SRA) under BioProject accession number PRJNA1491648. All statistical analyses and GSMM analyses were performed in the Python 3.11.5 environment using the following packages: scipy v1.11.1, scikit-learn v1.3.0, pandas v2.0.3, numpy v1.24.3, and cobra v0.31.1. Data visualization was performed using matplotlib v3.7.2 and seaborn v0.12.2.

## Results

### Information of the isolates and their lineage distribution

The 86 *C. jejuni* isolates collected in Korea belonged to 17 different CCs. Of the 17 CCs, nine had ≥ 2 isolates and the other eight were singletons. The most prevalent lineage was CC-21 (*n* = 36), followed by CC-354 (*n* = 8), CC-443 (*n* = 7), CC-464 (*n* = 6), CC-45 (*n* = 6), CC-607 (*n* = 5), CC-658 (*n *= 4), CC-5229 (*n* = 3), and CC-48 (*n* = 3). Most isolates were obtained from chicken (*n* = 70, 81%), and the remaining isolates were from humans (*n* = 16, 19%).

### Lineage-specific clustering of GSMMs

**Fig. 1:**
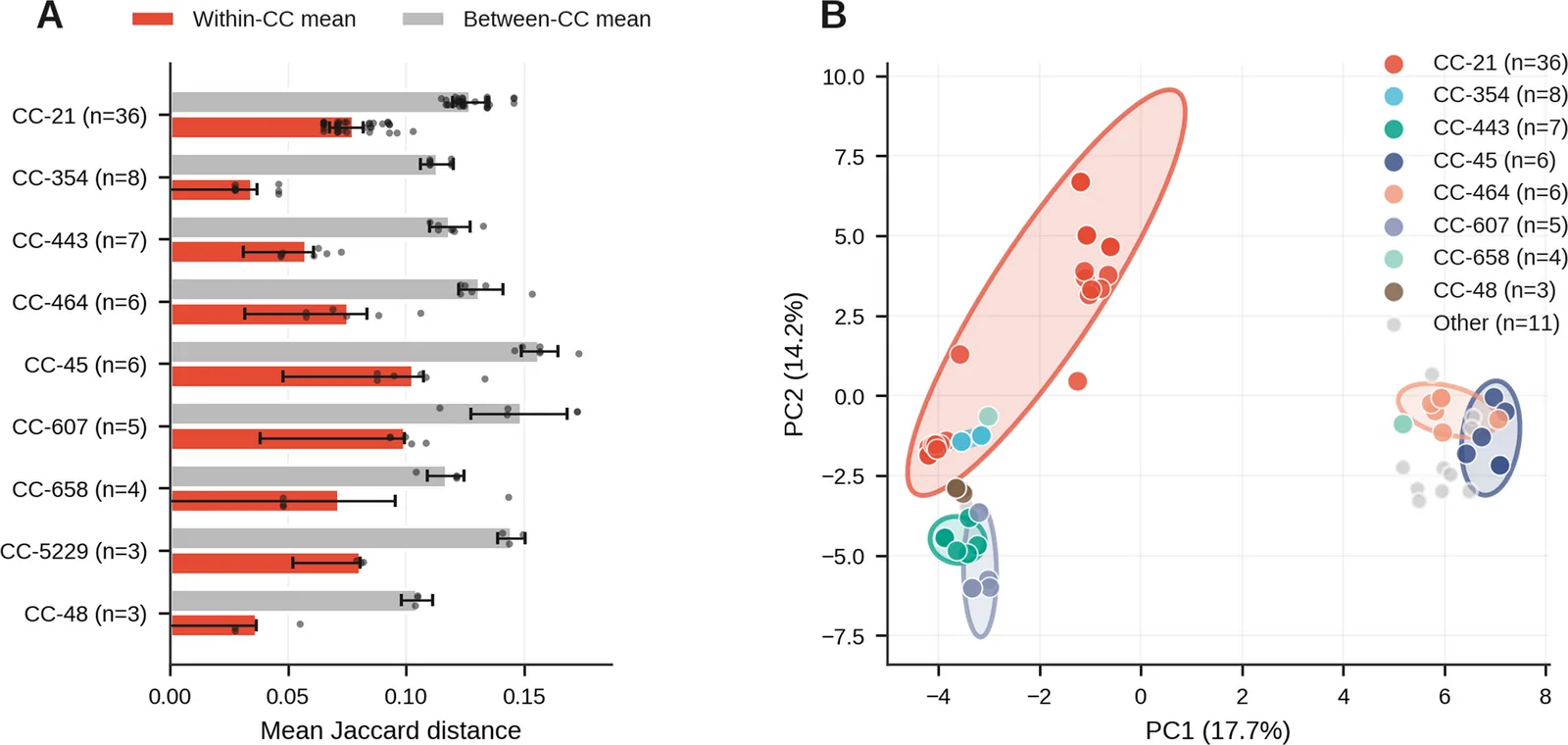
Lineage-specific clustering of GSMMs. **A** For each of the nine CCs with ≥ 2 isolates, the mean within-CC Jaccard distance (red) and mean between-CC Jaccard distance (gray); error bars show isolate-level bootstrap 95% confidence intervals, and each point is the mean distance of one isolate to the other isolates within or outside its CC. **B** Principal component analysis (PCA) of GSMM reaction composition for the 86 isolates, colored by the eight most prevalent CCs (remaining isolates in gray); ellipses show the 95% confidence regions. CC-level structure was significant by PERMANOVA (pseudo-*F* = 7.84, *P* < 0.001)

Strain-specific GSMMs were reconstructed for all 86 isolates. The models contained an average of 1505 reactions per isolate (range, 1391–1569) (Supplemental Table S3). The pan-reactome across all isolates consisted of 2313 unique reactions, including a core set of reactions shared by all isolates. Pairwise Jaccard distances were calculated for all 3655 strain pairs, and the distribution of distances across CC pairs was examined. Within-CC strain pairs showed a smaller median Jaccard distance than between-CC pairs (within-CC 0.082 *vs*. between-CC 0.130), corresponding to a 36.9% lower median within-CC distance. This clustering by CC was statistically significant (PERMANOVA pseudo-*F* = 7.84, *P* < 0.001; Mantel *r* = 0.54). In all nine CCs with ≥ 2 isolates, the mean within-CC distance was smaller than the mean between-CC distance (Fig. 1A). Consistent with the Jaccard distance-based analysis, principal component analysis (PCA) also showed clustering by CC (Fig. 1B). Isolate-level bootstrapping supported this separation: in all nine CCs, the 95% confidence intervals of the mean within- and between-CC Jaccard distances did not overlap (Fig. 1A; Supplemental Table S10). CheckM2 completeness was 99.95–100% for all isolates, and contamination was 0.16% or less for 85 of the 86 isolates; one isolate assigned to CC-45 showed 21.8% contamination. Individual quality metrics showed absolute Spearman correlations with reaction count of 0.41 or less (*r* = − 0.10 for completeness, + 0.06 for contamination, − 0.21 for contig count, + 0.27 for called cgMLST alleles, + 0.28 for GC content, and + 0.41 for the number of gap-filled reactions). After controlling for all of these metrics, the association between reaction-content distance and CC membership decreased from *r* = 0.54 to *r* = 0.45 but remained significant (partial Mantel *P* = 0.001). Excluding the contaminated isolate gave the same result (*r* = 0.44, *P* = 0.001; within-CC distances smaller than between-CC distances in all nine CCs in both analyses; Supplemental Table S5).

### GSMM and in vitro phenotypes were weakly correlated

We next examined whether the lineage-specific signals observed at the GSMM level are reflected in in vitro phenotypes. Phenotypes were quantified as the background-corrected 24 h fold change, calculated as the fold increase in OD in the presence of each substrate relative to the no-substrate control for each strain-substrate combination. Values were standardized as substrate-specific *z*-scores across all strains, where positive and negative values indicate above- and below-average growth, respectively. Euclidean distances were then calculated between the seven-substrate *z*-score vectors for the 86 strains with both GSMM and in vitro phenotype data.

**Fig. 2:**
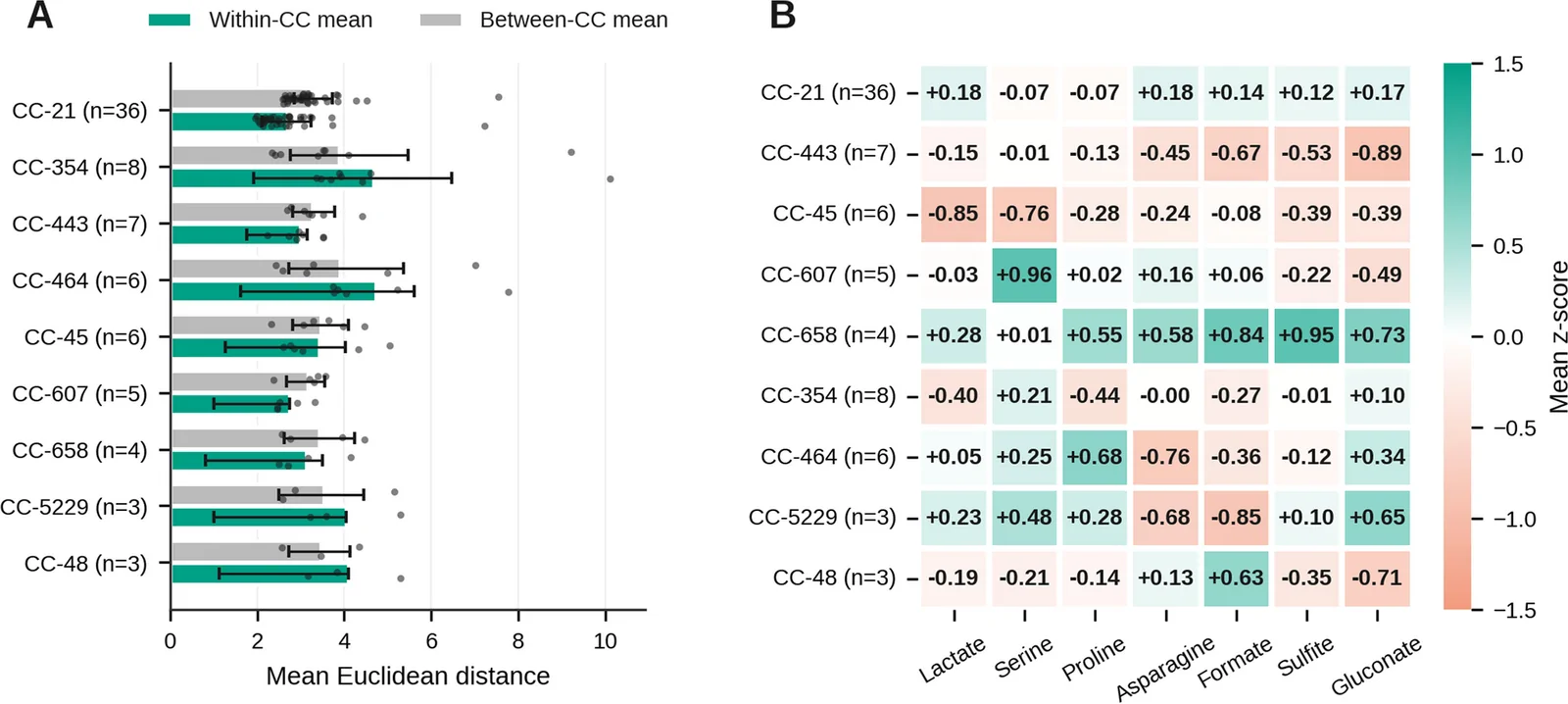
In vitro substrate utilization phenotypes across CCs. **A** For each of the nine CCs with ≥ 2 isolates, the mean within-CC Euclidean distance (green) and mean between-CC Euclidean distance (gray); error bars show isolate-level bootstrap 95% confidence intervals, and each point is the mean distance of one isolate to the other isolates within or outside its CC. **B** Mean *z*-scores for each substrate by CC. Numbers in each cell indicate the mean *z*-score. CC-level structure was not significant by PERMANOVA (pseudo-*F* = 1.00, *P* = 0.42)

Consistent with the GSMM analysis, the median within-CC distance was smaller than the between-CC distance (within-CC 2.50 *vs*. between-CC 3.09) in the in vitro phenotypes, but this difference was not statistically significant (PERMANOVA pseudo-*F* = 1.00, *P* = 0.42). Within-CC distances were smaller than between-CC distances in only five of the nine CCs for the in vitro phenotypes, compared with all nine CCs in the GSMM analysis (Fig. 2A). GSMM and in vitro distances were only weakly correlated (Mantel *r* = 0.13, *P* = 0.03). Among the five lineages with smaller within- than between-CC distances, some CCs showed distinct *z*-score profiles (Fig. 2B). For example, CC-443 showed negative *z*-scores across all substrates (formate, z = − 0.67; gluconate, z = − 0.89; sulfite, z = − 0.53; asparagine, z = − 0.45), whereas CC-658 showed positive *z*-scores across all substrates, particularly for sulfite (z = + 0.95), formate (z = + 0.84), and gluconate (z = + 0.73) (Fig. 2B). In contrast to the GSMM distances, the bootstrap confidence intervals of the within- and between-CC phenotype distances overlapped for all nine CCs (Fig. 2A; Supplemental Table S11). The substrate-specific *z*-scores used in this analysis have been added to the fold-change data in Supplemental Table S2.

### GSMM reaction differences associated with the distinct phenotypes of CC-443 and CC-658

**Fig. 3:**
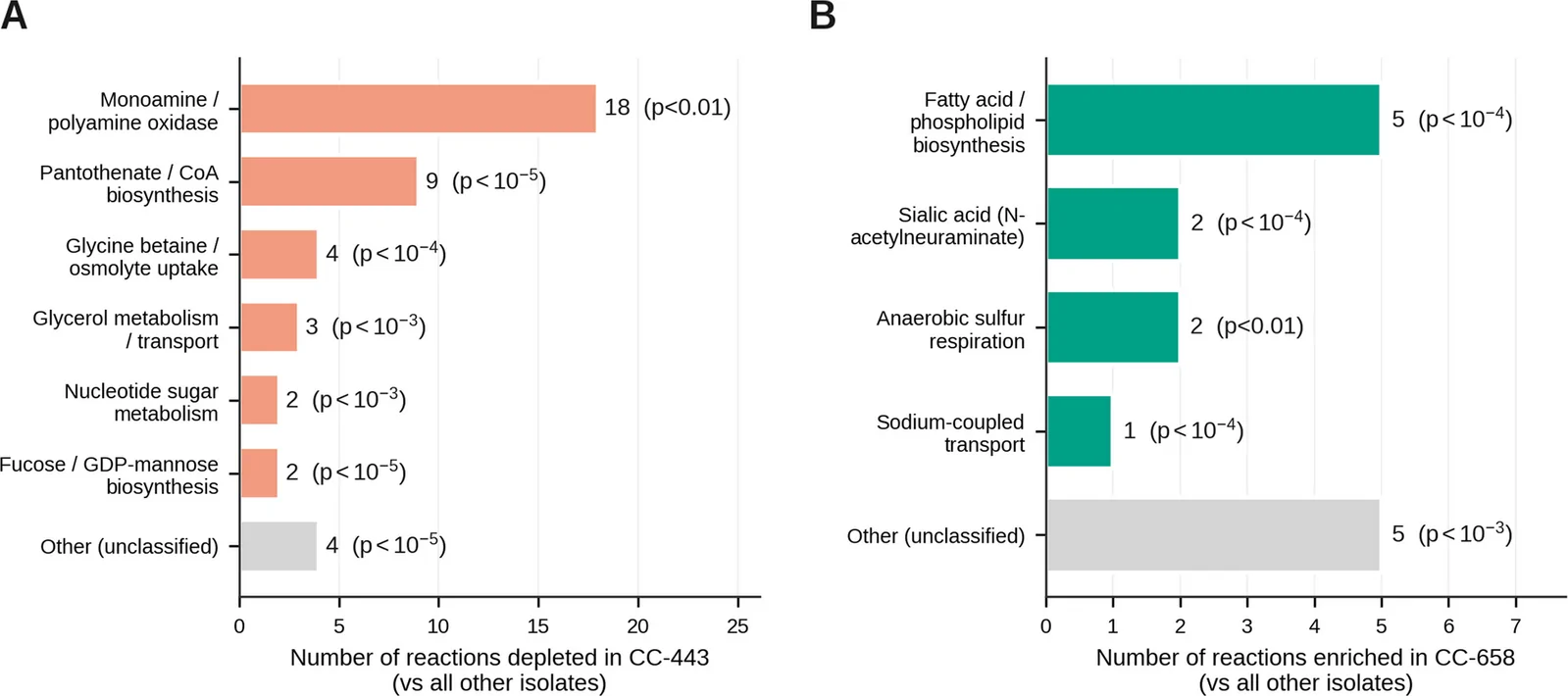
Lineage-associated reactions in CC-443 and CC-658. Reactions differing between each focal CC and all other isolates were grouped. The value beside each bar gives the number of reactions and the smallest nominal reaction-level Fisher’s exact test *P*-value within that category; category-level significance was not tested. Reaction-level nominal and FDR-adjusted results are given in Supplemental Table S6, and the direct comparison between the two lineages in Supplemental Table S7. **A** Reactions depleted in CC-443 (42 across six categories). **B** Reactions enriched in CC-658 (15 across four categories)

Because these two lineages were selected after inspection of the *z*-score profiles, the following comparison is exploratory. Given their distinct *z*-score profiles, we next examined the GSMM reaction content of CC-443 and CC-658 (Fig. 3). CC-443 lacked reactions across several functional categories, most notably the monoamine/polyamine oxidase family and pantothenate/CoA biosynthesis (Fig. 3A), whereas CC-658 was enriched in fatty acid/phospholipid biosynthesis and anaerobic sulfur respiration (Fig. 3B). These contrasts were computed for each CC against all other isolates; of the reactions identified at a nominal *P* < 0.01, 16 of 42 (CC-443) and 4 of 15 (CC-658) remained significant after FDR correction across all 2313 reactions (Supplemental Table S6). A direct comparison between the two lineages gave consistent results at the nominal level, including for the anaerobic sulfur respiration reactions (Fisher’s exact *P* = 0.024); with seven and four isolates in the two lineages, no reaction reached significance after FDR correction across all reactions tested, so this comparison is reported as exploratory (Supplemental Table S7). Lineage-associated reactions were not restricted to these two lineages: after FDR correction, all nine CCs except CC-48 (*n* = 3) carried reactions that were depleted or enriched relative to the remaining isolates. The affected pathways differed by lineage. Reactions annotated to aromatic compound degradation were enriched in CC-21 but depleted in CC-45 and CC-464. Among the depleted reactions of CC-443 the most frequent category was pantothenate and CoA biosynthesis, among those enriched in CC-354 it was tyrosine metabolism, and reactions annotated to glutathione and sulfur metabolism were among those enriched in CC-45. Counts and representative pathways for every CC are given in Supplemental Table S9. CC-658 comprised three chicken isolates collected in 2023 and one human isolate collected in 2020, whereas CC-443 consisted entirely of chicken isolates (Supplemental Table S1). The three chicken isolates of CC-658 had identical reaction content, and the human isolate differed from them at 229 reactions. When the comparison was restricted to chicken isolates, the anaerobic sulfur respiration reactions were present in all three CC-658 isolates and absent from all seven CC-443 isolates (100% vs. 0%; Fisher’s exact *P* = 0.008), indicating that this difference is not explained by isolation source. Collection year could not be separated from lineage, because the CC-658 chicken isolates were collected in 2023 and the CC-443 isolates in 2015–2018.

**Fig. 4:**
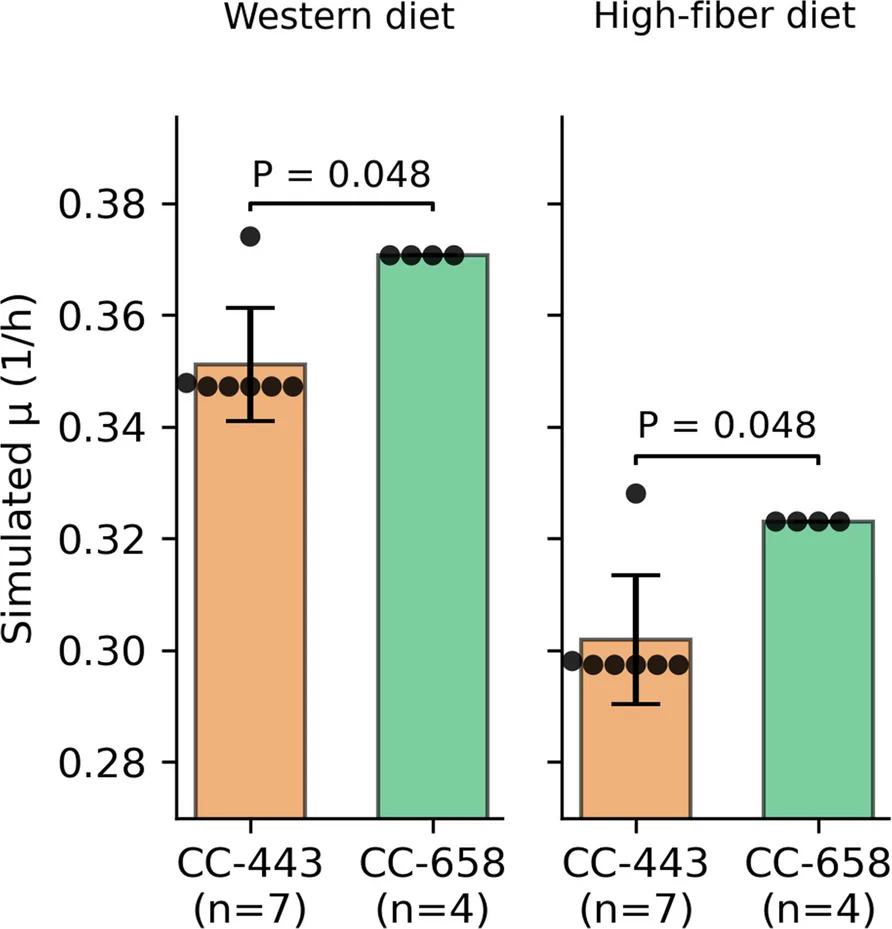
Predicted growth rates of CC-443 and CC-658. Bar plots show the mean ± SD of simulated growth rates (*μ*, 1/h) for CC-443 (*n* = 7, orange) and CC-658 (*n* = 4, green). Black dots represent individual isolates. Left: Western diet. Right: high-fiber diet. All four CC-658 models gave identical predicted values, hence SD = 0. Differences were assessed by a two-sided exact permutation test after rounding to ten decimal places to resolve ties (*P* = 0.048 for both diets); per-strain values are given in Supplemental Table S8

To examine whether these reaction differences affect growth, we simulated growth rates (*μ*, 1/h) for both lineages under two gut-mimicking dietary conditions (Western and high-fiber diet) as described elsewhere [24]. *μ* was obtained by FBA for the GSMM of each strain, and the mean *μ* values were compared between the two lineages. Because the direction of this comparison was suggested by the preceding results rather than specified in advance, a two-sided test was used. Under both dietary conditions, CC-443 (*n* = 7) exhibited consistently lower mean *μ* values than CC-658 (*n* = 4) (Fig. 4). Under the Western diet, the mean growth rates were 0.351 ± 0.010 for CC-443 and 0.371 ± 0.000 for CC-658 (exact *P* = 0.048). The same directional difference was consistently observed under the high-fiber diet, with mean *μ* values of 0.302 ± 0.012 for CC-443 and 0.323 ± 0.000 for CC-658 (exact *P* = 0.048). All four CC-658 models yielded identical predicted growth rates under both diets, so the standard deviation was zero, and five of the seven CC-443 models also converged on a single value. The reaction-content differences among these models did not change the maximum biomass production predicted under the simulated conditions. The effect size could not be estimated reliably under these conditions: in 9.4% of bootstrap resamples the pooled standard deviation fell below 1e − 12 and the estimate was treated as undefined, and across the remaining resamples the conditional 95% interval remained very wide (Cohen’s *d* = − 2.37 and − 2.25; Supplemental Table S8). The nominal significance of the permutation test should therefore be interpreted with caution, and the difference is reported primarily as a consistent direction. When the comparison was restricted to chicken isolates, the difference was not significant (exact *P* = 0.142 for both diets) (Supplemental Table S8).

## Discussion

GSMMs are systems biology models that reconstruct an organism’s metabolic capabilities by integrating genomic information with intracellular biochemical reactions, enzymes, and metabolic pathways in a computational framework, enabling quantitative simulation of metabolic phenotypes [25]. These models allow mathematical simulation of metabolic processes across diverse organisms, including archaea, bacteria, and eukaryotes [5]. Furthermore, GSMMs can be applied to biological discovery, such as predicting metabolic activities across microbial communities, as well as identifying potential drug targets in microbial pathogens [26].

In this study, rather than relying on the conventional approach based on a single reference strain [10–12], we investigated strain-level metabolic heterogeneity in 86 Korean *C. jejuni* isolates by integrating gapseq-based GSMM reconstruction with in vitro substrate utilization phenotypes. The central finding of this study is that CCs can be a factor in shaping GSMM reaction composition.

Clear lineage clustering was observed in GSMMs, with within-CC distances smaller than between-CC distances across all nine CCs. In the in vitro phenotypes, within-CC distances were smaller than between-CC distances in only five of the nine CCs, although the difference was not statistically significant. The GSMM and in vitro distances were only weakly correlated, and a similar weak correspondence between GSMM predictions and measured phenotypes was reported in clinical *P. aeruginosa* isolates [27]. Substrate utilization additionally reflects transcriptional regulation, protein expression, and assay conditions, none of which is captured by reaction content alone, which may contribute to this limited correspondence.

Despite this weak correspondence, the two lineages provided a potential genotype–phenotype link. In CC-658, the anaerobic sulfur respiration reactions (hydrogen sulfide and thiosulfate oxidoreductases) matched its high in vitro sulfite *z*-score of + 0.95. Conversely, the reactions depleted in CC-443, including the pantothenate/CoA biosynthesis pathway (which contributes to the tricarboxylic acid (TCA) cycle) [28] and the monoamine/polyamine oxidase family [29], were consistent with its uniformly low *z*-scores. In silico growth simulations showed the same direction, with lower predicted growth for CC-443 than for CC-658 under both diets. The difference was nominally significant in the exact permutation test. However, the eleven models gave only four distinct predicted values under each diet, so we regard this result as descriptive rather than confirmatory. The depletion of pantothenate/CoA biosynthesis in CC-443 is also consistent with established *C. jejuni* biology, as variation at the panBCD vitamin B5 biosynthesis locus has been associated with host-associated differentiation in this species [30]. Because vitamin B5 dependence was not tested experimentally here, this observation is described as consistent with, rather than demonstrating, such a mechanism.

This study has several limitations. First, all isolates originated from a single country (Korea) and were predominantly from chicken (81%), limiting the generalizability of the findings across countries and hosts. Second, the seven-substrate panel did not include mucin-derived sugars, leaving some GSMM predictions untested in vitro. Third, automated GSMM reconstruction depends on annotation databases, whose biases may affect reaction content. Fourth, some lineages had few isolates (CC-658, *n* = 4; CC-443, *n* = 7), limiting statistical power in the in silico growth simulation analysis. Finally, several lineage-enriched reactions, including sulfur respiration in CC-658, did not survive FDR correction and are considered candidate observations. Confirming these predictions will require enzyme assays, mutational studies, or in vivo colonization experiments. Moreover, the three chicken isolates of CC-658 had identical reaction content and identical predicted growth rates, so the effective number of independent observations in this lineage is smaller than the nominal sample size. Restricting the comparison to chicken isolates removed isolation source as an explanation, but collection year remained aligned with lineage (CC-658 chicken isolates, 2023; CC-443 isolates, 2015–2018), so a contribution of collection year cannot be excluded. Finally, the comparison of CC-443 and CC-658 was exploratory, as these lineages were selected after inspection of the phenotype profiles, and the corresponding results should be regarded as hypothesis-generating.

## Conclusions

We reconstructed GSMMs for 86 Korean *C. jejuni* isolates and found lineage clustering, with within-CC distances smaller than between-CC distances across all nine CCs, indicating that CC can be a factor in shaping GSMM reaction composition. This clustering was weaker in the in vitro phenotypes, and the GSMM and in vitro distances were only weakly correlated. However, the two lineages provided a potential genotype–phenotype link: CC-443 showed uniformly low *z*-scores and was depleted in several reactions including pantothenate/CoA biosynthesis, whereas CC-658 was enriched in anaerobic sulfur respiration, whose reactions matched its high sulfite *z*-score of + 0.95. In silico growth simulations showed that these reaction differences were accompanied by consistently lower predicted growth for CC-443, although the near-identical models within each lineage limit the strength of this comparison. These findings indicate that genome-scale metabolic modeling of *C. jejuni* should account for lineage-level diversity rather than relying on a single reference strain.

## Supplementary Information

Additional file 1: Supplementary Tables S1–S11.

## Data Availability

The datasets generated and analyzed during this study are provided in the Supplementary Tables. The whole-genome sequencing reads of the 86 isolates are available in the NCBI Sequence Read Archive under BioProject accession number PRJNA1491648. The analysis pipeline and figure-generation scripts are available from the GitHub repository: https://github.com/Jeong-Ih-Shin/cjejuni-gem-lineage-. The 86 strain-specific genome-scale metabolic models (SBML format) are available from Zenodo (https://doi.org/10.5281/zenodo.21886583).

## Abbreviations

CC: Clonal complex
CV: Coefficient of variation
FBA: Flux balance analysis
fc: Fold change
FDR: False discovery rate
GSMM: Genome-scale metabolic model
KEGG: Kyoto Encyclopedia of Genes and Genomes
MLST: Multilocus sequence typing
NCBI: National Center for Biotechnology Information
OD_600_: Optical density at 600 nm
PCA: Principal component analysis
PERMANOVA: Permutational multivariate analysis of variance
SBML: Systems Biology Markup Language
SD: Standard deviation
SRA: Sequence Read Archive
ST: Sequence type
TCA: Tricarboxylic acid
